# Inhibition of SARS Coronavirus Infection In Vitro with Clinically Approved Antiviral Drugs

**DOI:** 10.3201/eid1004.030458

**Published:** 2004-04

**Authors:** Emily L.C. Tan, Eng Eong Ooi, Chin-Yo Lin, Hwee Cheng Tan, Ai Ee Ling, Bing Lim, Lawrence W. Stanton

**Affiliations:** * Genome Institute of Singapore, Singapore; † National Environmental Agency, Singapore; ‡ Singapore General Hospital, Singapore

**Keywords:** In vitro, antivirals, agents, antiviral, antiviral agents, antiviral drugs, SARS virus, severe acute respiratory syndrome, coronavirus infection

## Abstract

Severe acute respiratory syndrome (SARS) is an infectious disease caused by a newly identified human coronavirus (SARS-CoV). Currently, no effective drug exists to treat SARS-CoV infection. In this study, we investigated whether a panel of commercially available antiviral drugs exhibit in vitro anti–SARS-CoV activity. A drug-screening assay that scores for virus-induced cytopathic effects on cultured cells was used. Tested were 19 clinically approved compounds from several major antiviral pharmacologic classes: nucleoside analogs, interferons, protease inhibitors, reverse transcriptase inhibitors, and neuraminidase inhibitors. Complete inhibition of cytopathic effects of SARS-CoV in culture was observed for interferon subtypes, β-1b, α-n1, α-n3, and human leukocyte interferon α. These findings support clinical testing of approved interferons for the treatment of SARS.

Severe acute respiratory syndrome (SARS) (*1*,*2*) is an infectious disease caused by a newly identified human coronavirus (SARS-CoV) (*3*,*4*). The disease can produce severe pneumonia with a reported fatal outcome of 15% to 20%. Currently, no effective drug exists to treat SARS-CoV infection (*5*). The urgency of the outbreak has led to the empiric use of broad- spectrum antibiotics and antiviral agents in affected patients in several countries (*6*–*12*). Intensive efforts are under way to gain more insight into the mechanisms of viral replication, in order to develop targeted antiviral therapies and vaccines. Developing effective and safe vaccines and chemotherapeutic agents against SARS CoV, however, may take years.

The recent epidemic has shown that knowledge is lacking regarding the clinical management and treatment of infected patients. Ribavirin (*6*–*12*), oseltamivir (*8*–*10*), foscarnet (*8*), intravenous immunoglobulin (*8*), and other agents have been used to treat patients. Preliminary results from in vitro testing indicate that ribavirin concentrations that inhibit other viruses sensitive to ribavirin do not inhibit replication or cell-to-cell spread of the SARS-CoV (*5*). However, the U.S. Centers for Disease Control and Prevention concluded that further in vitro testing of antiviral drugs on other coronavirus isolates and more information on the clinical outcome of patients treated with ribavirin or other antiviral drugs in controlled trials is needed (*5*).

The aim of this study was to investigate whether a panel of currently available antiviral agents exhibit in vitro anti–SARS-CoV activity. Three general antiviral strategies are generally found (*13*): (*1*) direct antiviral effects (*2*), inhibition of viral entry and replication at the cellular level by targeting virus-related processes, and (*3*) enhancement of host immune response. A total of 19 drugs approved for clinical use in the treatment of viral infections were tested in this study. They are representative compounds from major antiviral pharmacologic classes that are currently commercially available: nucleoside analogs, interferons, protease inhibitors, reverse transcriptase inhibitors and neuraminidase inhibitors.

A cell-based assay utilizing cytopathic endpoints (CPE) was set up using Vero E6 cells to screen these antiviral compounds. SARS-CoV has been shown to infect Vero E6 cells, an African green monkey kidney cell line (*3*), and this remains the only in vitro model of SARS-CoV infection. The initial screen was followed by a plaque reduction assay to determine the 50% effective concentration (EC_50_) of compounds showing positive results. These experiments allow rapid screening of commercially available antiviral agents, enabling those with in vitro evidence of activity to move expeditiously into clinical studies, since safety and pharmacokinetic information in humans is already available for other disease indications.

Here we report that certain interferon subtypes exhibit in vitro inhibitory activity against SARS-CoV and are candidates for follow-up studies in animal models and patients to determine their efficacy in vivo.

## Materials and Methods

### Selection and Preparation of Drugs

To rapidly identify a pharmacologic agent that could be used to treat SARS, a collection of antiviral drugs was tested against SARS-CoV, the etiologic agent of the atypical pneumonia. To investigate a wide spectrum of potential molecular targets, we decided to cover the entire pharmacologic range of commercially available antiviral agents, including agents not expected to be active against coronaviruses. Information on antiviral drugs provided here was obtained from prescribing information sheets or from communications with the manufacturer.

Nucleoside analogues are a diverse class of compounds; in general, they inhibit viral RNA or DNA polymerases or other enzymes, interfering with nucleic acid synthesis. In this study, the selected compounds that target DNA viruses such as herpes simplex virus (HSV) and varicella-zoster viruses (VZV) were acyclovir, ganciclovir, and foscarnet. Ribavirin has activity against a range of DNA and RNA viruses; in different cell lines, ED_50_ ranges from 1 to100 μg/mL. Antiretroviral (HIV) drugs include reverse transcriptase (RT) inhibitors and protease inhibitors. Selected HIV nucleoside RT inhibitors studied were zidovudine and lamivudine, while HIV protease inhibitors studied were indinavir, nelfinavir, and saquinavir. The third group of antivirals studied were the neuraminidase inhibitors, both commercially available preparations, zanamivir and oseltamivir were used in this study. Interferons were the next major class of antivirals studied. Various subtypes of interferon α (2a, 2b, n1, and n3, human leukocyte) and β (1a and 1b) were used. Amantadine, an old antiviral compound, was also studied. Different terms have been used to express antiviral activity, namely, EC_50_, 95% effective concentration (EC_95_), and 50% inhibitory concentration (IC_50_); Table 1 illustrates the range of activity against selected viruses.

**Table 1:** Commercially available antiviral agents tested, source and starting concentration

Antiviral agent	Source	Highest concentration tested	Inhibition of cytopathic effect (CIA_100_)
**Interferons**			
Interferon α-2a (Roferon)	Roche	100,000 IU/mL	No
Interferon α-2b (Intron A)	Schering-Plough	500,000 IU/mL	No
Interferon α-n1 (Wellferon)	GlaxoSmithKline	500,000 IU/mL	Yes
Interferon α-n3 (Alferon)	Hemispheryx	10,000 IU/mL	Yes
Interferon β-1a (Rebif)	Serono	500,000 IU/mL	No
Interferon β-1b (Betaferon)	Schering AG	100,000 IU/mL	Yes
**Nucleoside analogs**			
Acyclovir	Faulding	1,000 μg/ml	No
Cymevene (Ganciclovir)	Roche	50,000 μg/mL	No
Ribavirin	ICN Pharma	10,000 μg/mL	Yes
**Protease inhibitors**			
Indinavir (Crixivan)	Merck	100 μM	No
Nelfinavir (Viracept)	Roche	10,000 nM	No
Saquinavir (Fortovase)	Roche	10,000 nM	No
**Reverse transcriptase inhibitors**			
Lamivudine (Epivir)	GlaxoSmithKline	1,000 μM	No
Zidovudine (Retrovir)	GlaxoSmithKline	1,000 μg/mL	No
**Neuraminidase inhibitors**			
Oseltamivir (Tamiflu)	Roche	10,000 μM	No
Zanamivir (Relenza)	GlaxoSmithKline	1,000 μM	No
**Other**			
Amantadine (Symmetrel)	Novartis	1,000 μg/mL	No
Foscarnet (Foscavir)	AstraZeneca	8,000 μM	No

Tenfold dilutions of the drug were tested to cover a broad range of concentrations above and below inhibitory dosages as reported by the manufacturer for other viral-host combinations. Compounds already present in aqueous injections were made up to volume by using Hank’s buffered saline solution. For tablet and capsule formulations with soluble active ingredients, the outer coat was removed wherever applicable, and the preparation was ground in a mortar and pestle. The contents were dissolved in water, vortexed, and centrifuged thereafter at 3,000 *g*. The required volume was pipetted from the supernatant and diluted accordingly. When the active ingredients were insoluble in water (nelfinavir and saquinavir), the contents were dissolved in dimethylsulphoxide (DMSO); care was taken to ensure that the final concentration of DMSO in the dilutions would not exceed 1%. For plaque assays, fivefold drug dilutions were prepared by using growth media as specified below.

### SARS-CoV Production and Infection

Vero E6 cells (American Type Culture Collection, Manassas, VA) were propagated in 75 cm^2^ cell culture flasks in growth medium consisting of medium 199 (Sigma, St Louis, MO) supplemented with 10% fetal calf serum (FCS; Biological Industries, Kibbutz Beit Haemek, Israel). SARS-CoV 2003VA2774 (an isolate from a SARS patient in Singapore), which has been previously sequenced (*14*), was propagated in Vero E6 cells. Briefly, 2 mL of stock virus was added to a confluent monolayer of Vero E6 cells and incubated at 37°C in 5% CO_2_ for 1 h; 13 mL of medium 199 supplemented with 5% FCS was then added. The cultures were incubated at 37^o^C in 5% CO_2_, and the supernatant was harvested after 48 h; in >75% of cultures, inhibition of CPE (3+) in each well was observed with an inverted microscope. The supernatant was clarified at 2,500 rpm and then divided into aliquots, placed in cryovials, and stored at –80°C until use.

### Virus Handling and Titration

All virus culture and assays were carried out in the biosafety level-3 laboratory at the Environmental Health Institute, according to the conditions set out in Biosafety in Microbiological and Biomedical Laboratories (*15*). Virus titer in the frozen culture supernatant was determined by using a plaque assay. Briefly, 100 μL of virus in 10-fold serial dilution was added, in duplicates, to a monolayer of Vero E6 cells in a 24-well plate. After 1 h of incubation at 37°C in 5% CO_2_, the viral inoculum was aspirated, and 1 mL of carboxymethylcellulose overlay with medium 199, supplemented with 5% FCS, was added to each well. After 4 days of incubation, the cells were fixed with 10% formalin and stained with 2% crystal violet. The plaques were counted visually, and the virus titer in plaque-forming units per mL (PFU/mL) was calculated.

### Cytopathic Endpoint Assay

The protocol used was adapted from Al-Jabri et al. (*16*), and all drugs were tested in quadruplicate. Briefly, 100 μL of serial 10-fold dilutions of the drugs were incubated with 100 μL of Vero E6 cells, giving a final cell count of 20,000 cells per well in a 96-well plate. The incubation period was 1 h at 37°C in 5% CO_2_, except for the interferons, which were incubated overnight with the cells. Ten μL of virus at a concentration of 10,000 PFU/well was then added to each of the test wells. The plates were incubated at 37°C in 5%CO_2_ for 3 days and observed daily for CPE. The end point was the drug dilution that inhibited 100% of the CPE (CIA_100_) in quadruplicate wells. To determine cytotoxicity, 100 μL of serial 10-fold dilutions of the drugs was incubated with 100 μL of Vero E6 cells, giving a final cell count of 20,000 cells per well in a 96-well plate, without viral challenge. The plates were then incubated at 37°C in 5% CO_2_ for 3 days and examined for toxicity effects by using an inverted microscope.

### Plaque Reduction Assay

Trypsinized Vero E6 cells were resuspended in growth medium and preincubated with interferons (serial fivefold dilution) in quadruplicate wells in 24-well plates. The next day, the medium was aspirated, and 100 μL of virus was added to each well at a titer of 100 PFU/well. After incubation for 1 h, the virus inoculum was aspirated, and a carboxymethylcellulose overlay containing maintenance medium and the appropriate interferon concentration was added. After 4 days’ incubation, the plates were fixed and stained as described previously. The number of plaques was then counted visually, and the concentration of drug that inhibits 50% of plaques in each well (IC_50_) was determined. Results were plotted in Microsoft Excel, and a polynomial of order three was used to approximate the data and extrapolate IC_50_ and IC_95_ values.

## Results

### Cell-based Assay of SARS-CoV Infection

**Figure 1:**
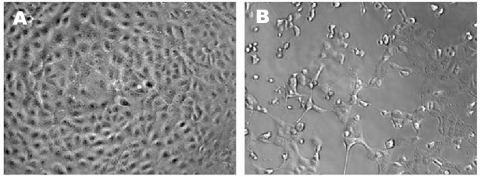
Microscopic appearance of control (a) and infected (b) Vero E6 cells demonstrating cytopathic effects.

High titers of infectious SARS-CoV, originally derived from a respiratory sample of a SARS patient, were propagated on Vero E6 cells. The CPE of SARS-CoV on Vero E6 was evident within 24 hours after infection (Figure 1). SARS-CoV–infected cells display a CPE characterized by the appearance of rounded cells and the destruction of the monolayer.

### Antiviral Drug Activity

A collection of 19 antiviral drugs was tested in the SARS-CoV CPE inhibition assay (Table 2). The set of drugs tested included seven interferons, five nucleoside analogs, three protease inhibitors, two RT inhibitors, and two neuraminidase inhibitors. Complete inhibition of the CPE was observed for four of the seven interferons in the initial screen when very high viral challenge of 10^4^ PFU/well and a high multiplicity of infection (MOI = 0.5) rate were used. Complete inhibition, expressed as CIA_100_, was observed for interferon β-1b (Betaferon) at 5,000 IU/mL, interferon α-n3 (Alferon) at 5,000 IU/mL, interferon α-n1 (Wellferon) at 250,000 IU/mL, and human leukocyte interferon α (Multiferon) at 500,000 IU/mL. Ribavirin also completely inhibited the CPE at 5,000 μg/mL (Table 3). None of the other drugs showed complete inhibition of CPE, even at the highest concentration of drug tested (Table 2).

**Table 2:** Examples of inhibitory concentrations of antiviral drugs against selected viruses^a^

Compound	IC_50_	Virus
Foscavir	50–800 μM	Cytomegalovirus
	5–443 μM	Herpes simplex mutants
Acyclovir	0.01–13.5 μg/ml	Herpes smplex virus and varicella-zoster virus
Cymevene	0.02–3.48 μg/mL	Laboratory strains or clinical isolates of cytomegalovirus
Ribavirin	1–25 μg/mL	Influenza
	25–100 μg/mL	HIV and other retroviruses
	3.2–50 μg/ml (MIC)	Herpes and poxviruses suppression
Lamivudine	0.0006–0.034 μg/mL	HIV
Zidovudine	0.003–0.013 μg/mL	HIV
Fortovase	1–30 nM	HIV
Viracept	7–196 nM (EC_95_)	HIV
Crixivan	25–100 nM	HIV
Relenza	0.005–16 μM	Influenza virus
Tamiflu	0.0008 μM–>35 μM	Influenza virus
Amantadine	0.1–25 (ED_50_)	Influenza virus

^a^IC_50_, 50% inhibitory concentration; EC_95_, 95% effective concentration; ED_50_, 50% effective dose.

**Table 3:** Complete inhibition of cytopathic effect (CIA_100_) at different virus titers

Virus load (PFU/well)	Ribavirin (μg/mL)	Wellferon (IU/mL)	Betaferon (IU/mL)	Alferon (IU/mL)
10,000	10,000	500,000	10,000	10,000
1,000	10,000	5,000	1,000	1,000
100	1,000	500	10	100

Rebif (IFN-β-1a) showed slight inhibition of CPE at 250,000 IU/mL, but the inhibition was not complete at the screening virus load of 10,000 PFU/well. Likewise, Roferon (IFN-α-2a) showed slight, incomplete inhibition at 50,000 IU/mL. Because the criteria for ascertaining anti-SARS-CoV activity in this screen were set at 100% inhibition of CPE, and as high doses of interferons may result in severe clinical side effects, we chose to conduct further evaluations only in the interferons that showed complete inhibition from initial screen, namely, Wellferon, Multiferon, Betaferon, and Alferon.

Based upon results of the primary screen, the four active interferons and ribavirin were retested at two lower viral challenges, 10^3^ and 10^2^ PFU/ well. All four drugs again showed inhibitory effect, although the CIA_100_ were dependent on viral loads (Table 3). At the lowest viral load the CIA_100_ were 5 IU/mL for both interferon β-1b (Betaferon) and human leukocyte interferon α (Multiferon); and 50 and 250 IU/mL for interferon α-n3 (Alferon) and interferon α-n1 (Wellferon), respectively. No cytotoxicity of the interferons was observed at or near inhibitory concentrations. Ribavirin showed inhibitory activity at all three viral loads, but only at high concentrations of the drug, 0.5–5 mg/mL. At high concentrations of ribavirin (0.2–1 mg/mL) cytotoxic effects were observed on VeroE6 cells, as has been reported for other cell types (*17*,*18*). As such, we consider ribavirin to be inactive against SARS-CoV.

**Figure 2:**
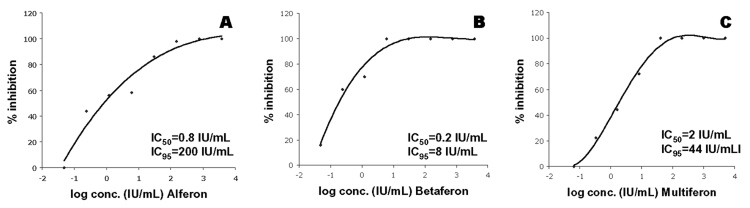
Dose-response curves for Alferon (a), Betaferon (b), and Multiferon (c) as determined by plaque reduction assays. IC_50_ (50% inhibitory concentration) and IC_95_ (95% inhibitory concentration) values were calculated by using the fitted functions describing the curves.

A plaque reduction assay format with 100 PFU of SARS-CoV (MOI = 0.0005) was conducted to determine the IC_50_ for Betaferon, Alferon, and Multiferon, the three compounds that showed greatest potency for inhibition of CPE. Additional supply was not available for testing interferon α-n1 (Wellferon), as production of this drug has been discontinued. Cells were preincubated for 15 h with fivefold dilutions of drug. Viral-induced plaques, which developed in 3 days, were counted to determine the inhibitory effect of the drugs at various concentrations. All three interferon preparations displayed a dose-dependent inhibition of SARS-CoV plaque formation in this assay (Figure 2). The IC_50_ and IC_95_ were determined to be 0.2 and 8 IU/mL for Betaferon, 0.8 and 200 IU/mL for Alferon, and 2 and 44 IU/mL for Multiferon.

## Discussion

Betaferon, Alferon, Multiferon, Wellferon, and ribavirin inhibited CPE in SARS-CoV–infected Vero E6 cells, in decreasing order of potency. Ribavirin, a drug widely used in initial efforts to manage SARS infections, inhibited CPE completely at 500–5,000 μg/mL at virus loads of 100–10,000 PFU per well. The concentration range observed is much higher than concentrations that inhibit other viruses (respiratory syncytial virus, ED_50_ 2–8 μg/mL, HIV or resistant strains of rhinovirus, 50–100 μg/mL), including viruses that were tested on Vero cells (West Nile virus, New York isolate 178 μg/mL, and Uganda isolate 41 μg/mL) (*19*). In addition, the CPE inhibitory concentrations obtained in this study were above the cytotoxic concentration range against Vero cells. The 50% cytotoxic dose (CD_50_) on various cell lines has been reported to be approximately 200–1000 μg/mL of ribavirin (*17*,*18*). We observed slight cytotoxicity by microscopic examination of the cells, making it difficult to accurately obtain in vitro efficacy data against SARS-CoV. It appears that due to the low activity of ribavirin in vitro, inhibitory doses may not be achievable clinically. It is possible that ribavirin would be more effective in combination with interferons. Combination therapy with ribavirin and interferon α has now become standard treatment for chronic hepatitis C (*20*–*22*). Additionally, we have tested the effect of ribavirin and Betaferon in combination (range of concentration of ribavirin, 1–100 μg/mL; range of concentration of Betaferon, 0.1–10 IU/mL). At 1,000 PFU, this combination did not demonstrate observable synergistic inhibitory effect against SARS-CoV.

This study describes in vitro activity of four interferon subtypes against the SARS-CoV. Interferons have been used as anticancer and antiviral agents, in particular, for treating hepatitis B and C infections. Various groups have reported the clinical benefit of intranasally administered interferon α in human volunteers before and after inoculation with non-SARS coronaviruses (*23*–*25*). The antiviral activity of interferons is mediated by direct effects on infected cells or by modulating an immune response (*26*). Interferons interact with specific surface cell receptors, leading to production of interferon-stimulated gene products such as 2′5′-oligoadenylate synthase and protein kinase PKR (*27*).

In SARS-CoV infection, a convenient starting point for the use of interferons against a SARS-CoV infection would be the usual clinical doses for the treatment of hepatitis B or C. Common clinical dosages for interferon α range from 3 to 5 million IU three times a week to 5 million IU daily. For interferon β, data regarding efficacy in the treatment of hepatitis C are conflicting, and interferon β (at doses of 3 to 6 million IU three times weekly) is usually only used in the treatment of infections in patients whose condition no longer responds to other therapies. Plasma levels of interferons administered through the subcutaneous route are usually low with correspondingly short half-lives. In view of their mechanism of action, absolute serum levels may not be meaningful as a measure of the biologic activity of interferons, compared to the induction of cellular products such as 2′5′ oligoadenylate synthase.

Interferon activity varies among different cell types (*28*,*29*), however. Specific interferon subtypes which inhibit SARS-CoV in Vero cells may not necessarily have the same effect in other cells; the converse may also be true—that those drugs that are negative in Vero cells may be effective in other cell types. We are currently identifying other in vitro models of SARS-CoV infection that will enable us to address cell-type specific drug effects. Also, interferon subtypes exhibited different activity against SARS-CoV in this study. The mechanism for the difference in activity is unknown. Among the products tested, the source of interferon and amount of glycosylation differ. Some preparations were derived from human lymphoblastoid or leukocyte cells, while others were recombinantly produced in *Escherichia coli* or mammalian cell culture. We do not know the importance of this observation with respect to possible antiviral mechanisms of the interferons against SARS-CoV or potential clinical implications of these differences.

This study describes rapid screening of commercially available compounds for extension into in vivo research. Evidence of activity and data from in vitro studies, however, cannot be easily correlated with clinical performance but rather present promising candidates for follow-up studies. Definite recommendations on anti–SARS-CoV activity of compounds in humans can only be made in the in vivo setting. In conclusion, interferon β-1b, α-n1, α-n3, and human leukocyte interferon α exhibit antiviral activity in an in vitro model and are potential drugs for in vivo research and clinical management of SARS-CoV infection.
